# SOCS1 regulates hepatic regenerative response and provides prognostic makers for acute obstructive cholangitis

**DOI:** 10.1038/s41598-017-09865-z

**Published:** 2017-08-25

**Authors:** Jianhua Yu, Weiguang Zhang, Hongwei Qian, Haijun Tang, Weiguo Lin, Baochun Lu

**Affiliations:** 1 Department of Hepatobiliary Surgery, Shaoxing People’s Hospital, Shaoxing Hospital of Zhejiang University, Shaoxing, China; 2 Department of Molecular Medicine and Clinical Laboratory, Shaoxing Second Hospital, Shaoxing, China

## Abstract

Acute obstructive cholangitis (AOC) is a common and severe infectious diseases that occurs in an obstructed biliary system. The suppressors of cytokine signaling (SOCS) family include well-known negative regulators of cytokine receptor signaling. However, few studies have been conducted to determine their function in AOC. In this study, we showed that SOCS1 expression aberrantly changed and was associated with AOC prognosis in rat models. Decreased SOCS1 expression enhances regenerative response after biliary drainage (BD) resulting from AOC by upregulating hepatocyte growth factor (HGF) signaling. To detect SOCS1 expression in the liver less invasively and to predict the prognosis for AOC after BD, miR-221 and miR-222 were investigated. Ectopic SOCS1 expression indirectly decreases miR-221/222 expression through Met *in vitro*. An inverse correlation between SOCS1 expression and miR-221/222 expression in liver tissue or in serum was verified in rats. Serum from AOC patients showed that lower expression of circulating miR-221/222 after endoscopic nasobiliary drainage was associated with delayed restoration of liver function. Our results showed that SOCS1 regulates hepatic regenerative response, and indirectly detecting downstream molecules, such as miR-221/222, may provide prognostic makers for AOC.

## Introduction

Acute obstructive cholangitis (AOC) is a common biliary tract infection with rapid progression that can have a poor prognosis. For AOC, complete biliary obstruction is the initiating factor, and secondary infection with a Gram-negative pathogen worsens the condition^1, 2^. Liver is the earliest organ involved and it is always injured seriously during AOC; the level of liver injury is a key factor affecting prognosis. Both cholestasis and lipopolysaccharide (LPS) are independent factors that induce liver injury, but AOC-induced liver injury shows different characteristics^3^. For example, in response to cholestasis-induced liver injury, hepatocytes will proliferate to compensate for their respective loss^4^. Although there are many studies on the regulatory mechanism of independent cholestasis- or LPS-induced liver injury^5–8^, understanding the hepatic response in AOC remains elusive.

The suppressors of cytokine signaling (SOCS) family includes SOCS1-7 and cytokine-inducible SH2-containing protein (CIS), and it is a family of well-known negative regulators of cytokine receptor signaling^9^. Many studies suggest that SOCS proteins play an important role in inflammatory responses, proliferation, apoptosis, and oncogenesis^10–12^. Although some SOCS proteins, such as SOCS1 and SOCS3, have been shown to play an important role in LPS-induced liver injury^13, 14^, few have shown involvement in cholestasis- or AOC-induced injury.

MicroRNAs (miRNA) are key negative regulators of gene expression in eukaryotes and there is strong evidence that miRNAs are involved in hepatic disease^15, 16^. MicroRNA-221 (miR-221) and microRNA-222 (miR-222), which have similar conserved sequences, are considered to be an important miRNA cluster in many human cancers^17–20^. Additionally, miR-221/222 expression can be enhanced by the activation of Met, which is a key growth factor receptor in cells^17, 21^. Although recent studies reported that miR-221/222 are involved in liver diseases, including liver fibrosis and tumorigenesis^22, 23^, their potential function during cholestasis diseases, such as AOC, is unclear.

Here, we showed that SOCS1 expression is aberrant in AOC and that it is associated with the AOC prognosis through regulation of the hepatic regenerative response in an animal model. Further research suggests that decreased SOCS1 expression enhances the regenerative response through upregulating hepatocyte growth factor (HGF) signaling. Because Met activation by growth factors can enhance miR-221 and miR-222 expression, we investigated the possibility that indirectly evaluation of SOCS1 expression levels by examining miR-221 and miR-222 expression may predict AOC patient prognosis. Our results suggest that SOCS1 regulates the hepatic regenerative response and that it is a novel prognostic maker during AOC.

## Results

### SOCS1 and SOCS3 expression is upregulated during acute obstructive cholangitis

Hepatic function was evaluated to compare the results obtained using our AOC animal model with results from previous studies^24, 25^. ALT, AST, and TBIL levels increased rapidly after BDL compared with those after the sham operation (Table. 1). ALT, AST, and TBIL levels were further increased by an infusion of LPS into the bile duct (AOC group), similar to the classical changes observed in AOC patients. However, intraperitoneal LPS infusion (LPS group) did not cause as much liver damage as in the AOC group. These results indicate that liver injury arose in our AOC animal model.

**Table 1:** Data of laboratory examination at 12 h after operation (n = 6).

	sham	LPS	BDL	AOC
TBIL(μmol/L)	9.72 ± 2.51	12.61 ± 4.63	43.83 ± 9.37^*,#^	72.12 ± 9.75^*,#,&^
ALT(IU/L)	60.50 ± 7.25	80.17 ± 7.75^*^	539.17 ± 89.69^*,#^	843.83 ± 84.05^*,#,&^
AST(IU/L)	84.17 ± 7.06	102.50 ± 14.27^*^	825.17 ± 88.69^*,#^	1440.17 ± 175.83^*,#,&^

TBIL, total bilirubin; ALT, alanine aminotransferase; AST, aspartate aminotransferase. Values are expressed as mean ± standard deviation.

^*^ *P* < 0.05, compared with sham group.

^#^ *P* < 0.05, compared with LPS group.

^&^ *P* < 0.05, compared with BDL group.

**Figure 1:**
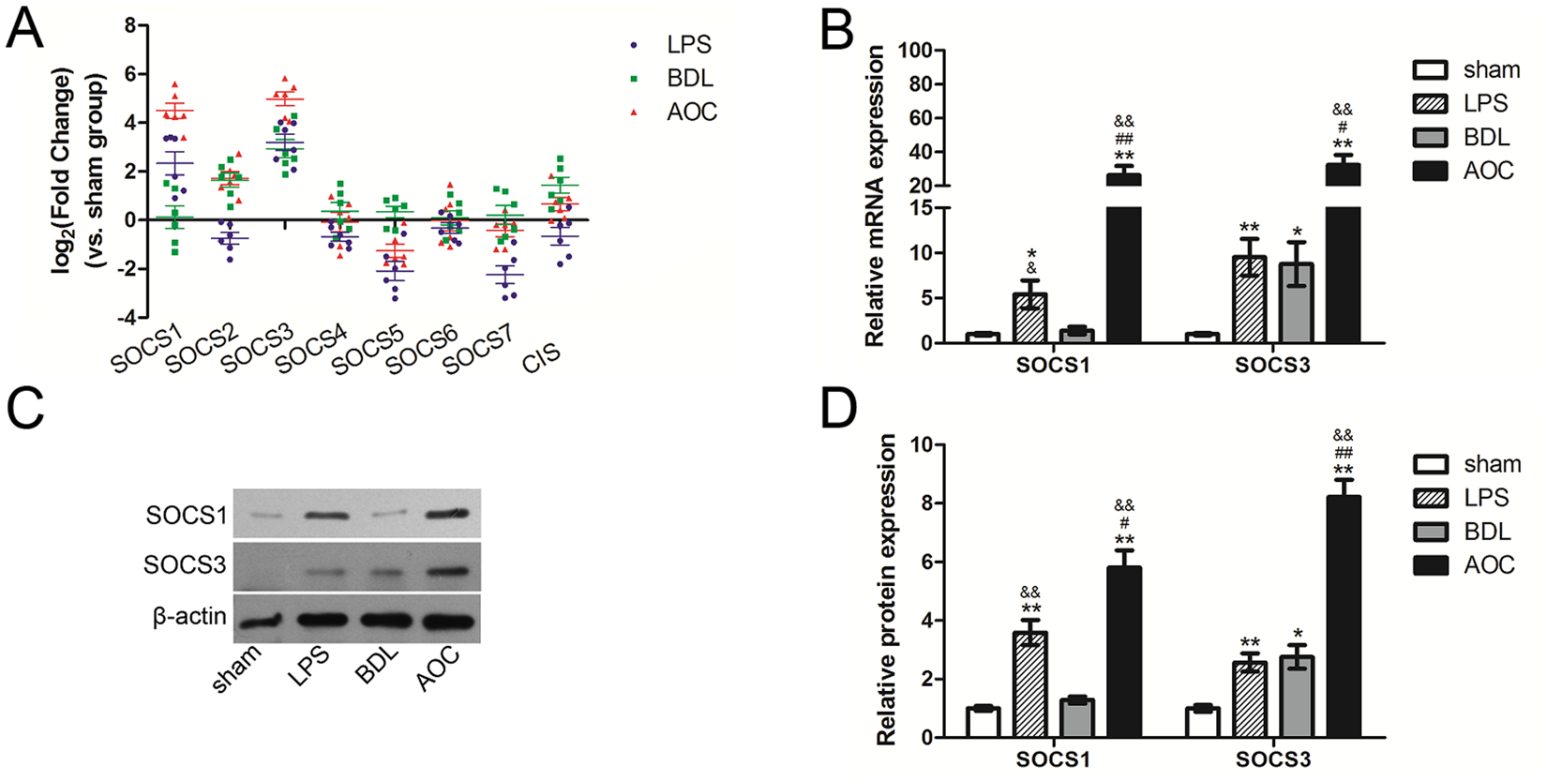
SOCS1 and SOCS3 expression is upregulated during acute obstructive cholangitis. (**A**) Real-time PCR was performed to examine the expression difference of SOCSs in liver tissues from different rat models. (**B**) Real-time PCR and western blot (**C** and **D**) were performed to examine the expression level of SOCS1 and SOCS3 in liver tissues from different rat models. Six rats were used in each group. *P < 0.05; **P < 0.01 versus sham group. ^#^ *P* < 0.05; ^##^ *P* < 0.01 versus LPS group. ^&^ *P* < 0.05; ^&&^ *P* < 0.01 versus BDL group.

To determine the correlation between SOCS proteins and AOC-induced liver injury, we compared the hepatic mRNA expression of eight SOCS family members. Both SOCS1 and SOCS3 were significantly upregulated in the AOC group compared with all other groups (Fig. 1A and B). Other SOCS proteins did not show a consistent and significant difference in the AOC group compared with the other three groups (Fig. 1A). Western blot analysis also confirmed the real-time PCR results (Fig. 1C and D).

### SOCS1 expression is associated with acute obstructive cholangitis prognosis

**Figure 2:**
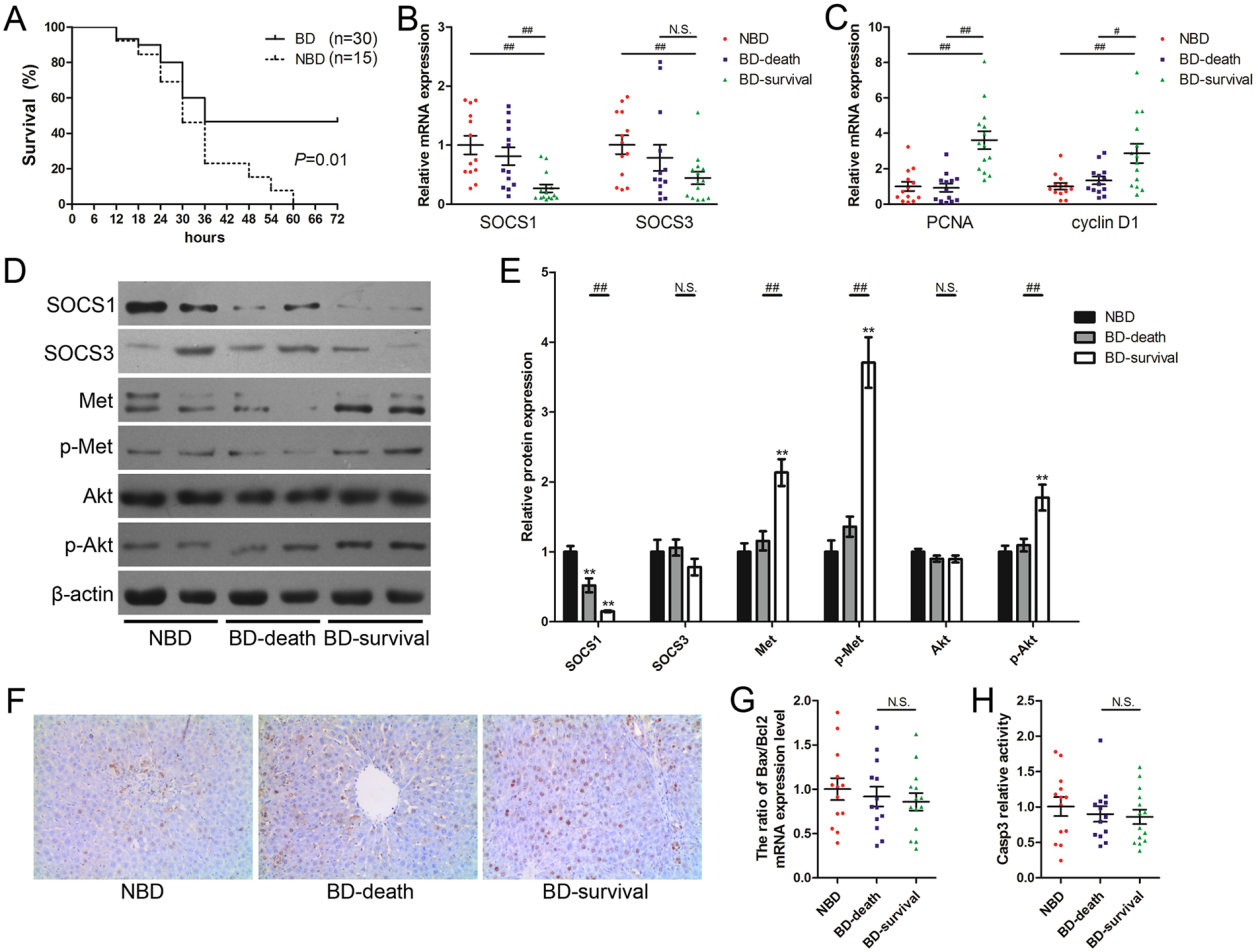
SOCS1 regulates hepatic regenerative response and is associated with acute obstructive cholangitis prognosis. (**A**) The survival rates of the AOC rats which received biliary drainage (BD group) or did not received biliary drainage (NBD group) at 12 h after LPS infusion. (**B**) Relative expression of SOCS1 and SOCS3 in liver tissues from AOC rats with different prognosis. (**C**) Relative expression of PCNA and cyclin D1 in liver tissues from AOC rats with different prognosis. (**D** and **E**) Determination of relative protein levels in liver tissues from AOC rats with different prognosis. (**F**) Immunohistochemical staining of PCNA in liver tissues from AOC rats with different prognosis (original magnification ×200). (**G** and **H**) Apoptotic response in liver tissues from AOC rats with different prognosis was evaluated by the ratio of Bax to Bcl2 mRNA expression levels (**G**) and relative activity of Caspase 3 (**H**). The liver samples were collected at 18 h after LPS infusion into the bile duct (5 rats were excluded at the time point because of premature death). NBD, the rats without biliary drainage, *n* = 13. BD-death, the rats with biliary drainage and died in the 72-h observation period after LPS infusion, *n* = 13. BD-survival, the rats with biliary drainage and survived in the 72-h observation period, *n* = 14. PCNA, proliferating cell nuclear antigen; Bax, BCL2-associated X protein; Bcl2, B-cell CLL/lymphoma 2; Casp3, Caspase 3. **P < 0.01 versus NBD group. ^#^ *P* < 0.05; ^##^ *P* < 0.01 significant difference between the two groups. N.S. = non-significant, between the two groups.

AOC key treatment points are timely drainage and relief of obstruction^2^. To study whether there was potential relevance between AOC prognosis and changes in SOCS1 and SOCS3 expression, the catheter sealing caps were removed and bile was allowed to flow out from the catheters 12 h after LPS infusion into the bile duct. As expected, biliary drainage significantly reduced rat mortality (Fig. 2A). At 18 h after LPS infusion into the bile duct (6 h after biliary drainage), hepatic SOCS1 and SOCS3 expression was examined. Interestingly, the surviving rats with biliary drainage had significantly lower SOCS1 expression than rats that did not undergo biliary drainage or that underwent biliary drainage but died (Fig. 2B and D). However, SOCS3 expression did not show a significant correlation with the prognosis of AOC animals (Fig. 2B and D).

### Regenerative response during biliary drainage determines acute obstructive cholangitis prognosis

Regeneration and apoptosis are opposite processes that both occur during a hepatic response. Results of their contrasting effects will determine the effect on liver injury. Both regenerative and apoptotic responses were evaluated in surviving rats and in rats that died. Both proliferating cell nuclear antigen (PCNA) and cyclin D1 mRNA levels in the surviving group were significantly higher than those in the groups of rats that died (Fig. 2C). Immunohistochemistry also confirmed that PCNA protein level were higher in the rats that survived than in those that died (Fig. 2F).

Caspase 3 activity and the ratio of Bax to Bcl2 are often used to evaluate apoptotic response^26^. Neither caspase 3 activity nor the ratio of Bax to Bcl2 showed a remarkable difference between rats that survived and those that died (Fig. 2G and H). These results indicate that regenerative response during biliary drainage, rather than apoptotic response, should be the decisive factor for AOC prognosis.

### Decreased SOCS1 expression enhances regenerative response after biliary drainage of acute obstructive cholangitis by strengthening HGF signaling

**Figure 3:**
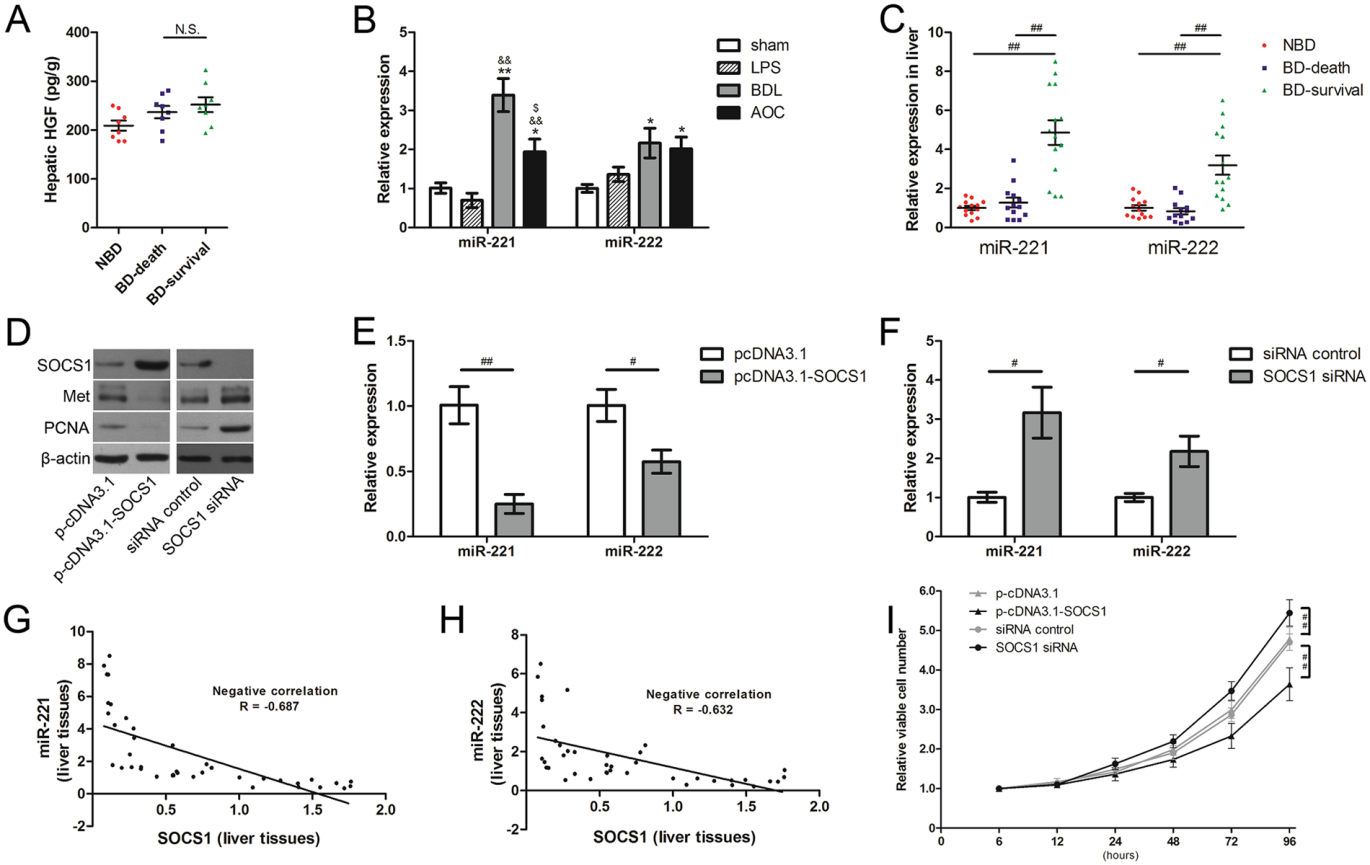
miR-221/222 expression is modulated by SOCS1/Met axis and is associated with acute obstructive cholangitis prognosis. (**A**) Determination of HGF levels in liver tissues from AOC rats with different prognosis. (**B**) Relative expression of miR-221 and miR-222 in liver tissues from different rat models. *n* = 6 each group, *P < 0.05; **P < 0.01 versus sham group. ^&&^ *P* < 0.01 versus LPS group. ^$^ *P* < 0.05 versus BDL group. (**C**) Relative expression of miR-221 and miR-222 in liver tissues from AOC rats with different prognosis. (**D**) Determination of the relationship between SOCS1 and Met protein levels by western blot analysis. (**E** and **F**) Determination of relative miR-221/222 levels under the condition of aberrant SOCS1 expression *in vitro*, three assays were performed. (**G** and **H**) Correlation coefficients (R) between the mRNA levels of SOCS1 and miR-221/222 were respectively calculated in liver tissue samples from AOC rats. (**I**) Cell proliferation assays released that silencing of SOCS1 significantly promotes proliferation of BRL cells *in vitro*. NBD, the rats without biliary drainage. BD-death, the rats with biliary drainage and died in the 72-h observation period after LPS infusion. BD-survival, the rats with biliary drainage and survived in the 72-h observation period. ^#^ *P* < 0.05; ^##^ *P* < 0.01; significant difference between the two groups. N.S. = non-significant, between the two groups.

HGF plays a key role during liver regeneration^27^ and HGF signaling can be strengthened in SOCS1-deficient hepatocytes^28^. Our results showed that hepatic HGF levels were not significantly different between rats that survived and those that died (Fig. 3A). However, Western blots showed that HGF downstream signaling molecules, including Met and AKT, had a significant increase in phosphorylation in rats that survived compared with the other groups (Fig. 2D and E). Western blot results also revealed an inverse correlation between SOCS1 and phospho-Met or phospho-AKT (Fig. 2D and E). This indicates that the strengthened HGF signaling, which was induced by decreased SOCS1 expression, might be attributable to the intensive regenerative response after BD.

### miR-221/222 expression is aberrant in the liver and is associated with acute obstructive cholangitis prognosis

Previous studies reported that Met is able to enhance miR-221 and miR-222 expression^17, 21^. Thus, we examined miR-221 and miR-222 expression in our study. At 6 h after operation, we found that miR-221 increased significantly because of the obstructed biliary tract (BDL group), while there was a mild decrease in response to LPS infusion (AOC group) (Fig. 3B). Meanwhile, miR-222 increased significantly in both BDL and AOC groups (Fig. 3B). We also examined miR-221/222 levels after BD in liver tissue from AOC rats. Rats with BD that survived had a significantly higher miR-221 and miR-222 level in the liver tissue compared with rats that died or without BD (Fig. 3C).

### SOCS1/Met axis modulates miR-221/222 expression in hepatocytes

To confirm that miR-221/222 expression can also be regulated by SOCS1/Met axis in hepatocytes, we performed an experiment *in vitro*. We found that SOCS1 overexpression in BRL cells reduces Met expression (Fig. 3D) and induces a dramatic decrease in miR-221 and miR-222 expression (Fig. 3E). As expected, SOCS1 RNA interference knockdown in rat hepatocytes showed a prominent increase of miR-221 and miR-222 expression (Fig. 3F).

*In vivo*, we used Pearson’s correlation analysis to analyze the potential correlation between miR-221/222 expression and SOCS1 mRNA expression in liver tissue. Both miR-221 and miR-222 had a statistically significant inverse correlation with SOCS1 (R = −0.687 and −0.632, respectively; Fig. 3G and H). This suggests that miR-221/222 expression in the liver may represent that of SOCS1 and indirectly reflect AOC prognosis.

### Circulating miR-221 and miR-222 are potential biomarkers to predict acute obstructive cholangitis prognosis

**Figure 4:**
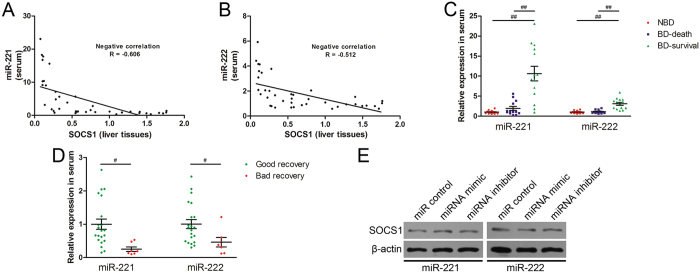
Circulating miR-221 and miR-222 are potential biomarkers to predict acute obstructive cholangitis prognosis. (**A** and **B**) Correlation coefficients (R) between the mRNA levels of SOCS1 in liver tissues and miR-221/222 in serum samples from AOC rats were calculated. (**C**) Relative expression of miR-221 and miR-222 in serum samples from AOC rats with different prognosis. NBD, the rats without biliary drainage. BD-death, the rats with biliary drainage and died in the 72-h observation period after LPS infusion. BD-survival, the rats with biliary drainage and survived in the 72-h observation period. ^##^ *P* < 0.01; significant difference between the two groups. (**D**) Relative expression of miR-221 and miR-222 in serum samples from AOC patients with different prognosis. The AOC patients who had decreasing TBIL after ENBD were defined as the “good recovery” group. Inversely, the patients who had increasing TBIL after ENBD were defined as the “bad recovery” group. ^#^ *P* < 0.05 significant difference between the two groups. (**E**) Determination of SOCS1 protein levels under the condition of aberrant miR-221/222 expression *in vitro*.

Circulating miRNA is easy to detect, and we examined circulating miR-221 and miR-222 levels in rat serum. We found that circulating miR-221 and miR-222 levels also were inversely correlated with SOCS1 expression in the liver (R = −0.606 and −0.512, respectively; Fig. 4A and B). Similar to miR-221/222 in liver tissues, circulating miR-221/222 in rats with BD that survived was also higher than in rats that died or without BD (Fig. 4C). This suggests that circulating miR-221 and miR-222 are potential biomarkers to predict the AOC prognosis in the rat.

To investigate whether circulating miR-221 and miR-222 had a similar function during the process of AOC in humans, serum samples from AOC patients were examined. Serum from 28 patients who had Charcot triad with high TBIL (100–200 μmol/L) and underwent endoscopic nasobiliary drainage (ENBD) were included in our study. We examined miR-221/222 levels in the serum collected 6 hours after ENBD. Three days after ENBD, liver function tests were performed in all patients. According to the TBIL and DBIL levels, the patients whose liver function tests showed increasing TBIL were defined as the “bad recovery” group (n = 7, Table 2), while the patients who had decreasing TBIL were defined as the “good recovery” group (n = 21, Table 2). As shown in Table 2, there was no difference in general characteristics between the two groups, such as age, gender, body mass index (BMI), with or without tumor and received antibiotics. The differences in ALT, AST, TBIL or DBIL between the two groups before ENBD were also no significant (Table 2). Interestingly, comparing the results between two groups, both miR-221 and miR-222 in the bad recovery group were significantly lower than those in the good recovery group (Fig. 4D). This indicates that circulating miR-221 and miR-222 levels are associated with AOC prognosis in rats and in humans.

**Table 2:** Characteristics and liver function of the AOC patients who underwent endoscopic nasobiliary drainage (ENBD).

Characteristics		Good Recovery (n = 21)	Bad Recovery (n = 7)	*P*
Age (yr)		64.24 ± 12.35	63.29 ± 11.66	0.864
Gender (Male/Female)		12/9	5/2	0.503
BMI		25.61 ± 2.66	26.13 ± 2.64	0.668
Tumor (Yes/No)		7/14	4/3	0.264
Received antibiotics (Yes/No)		21/0	7/0	NA
ALT (IU/L)	Before ENBD	211.83 ± 132.65	123.10 ± 94.97	0.116
	After ENBD	108.45 ± 70.36	60.13 ± 33.15	0.094
AST (IU/L)	Before ENBD	164.26 ± 98.51	112.57 ± 61.64	0.206
	After ENBD	56.83 ± 51.29	66.11 ± 34.94	0.661
TBIL (μmol/L)	Before ENBD	137.41 ± 31.83	132.09 ± 30.92	0.702
	After ENBD	63.99 ± 39.25	176.63 ± 58.39	**<0.001**
DBIL (μmol/L)	Before ENBD	95.18 ± 25.27	92.27 ± 26.64	0.797
	After ENBD	42.77 ± 27.51	126.20 ± 42.96	**<0.001**
CRP (mg/L)	Before ENBD	78.56 ± 32.98	77.35 ± 22.14	0.931
	After ENBD	73.93 ± 19.11	74.89 ± 17.56	0.911

Good recovery, the AOC patients who had decreasing TBIL after ENBD. Bad recovery, the patients who had increasing TBIL after ENBD. ENBD, endoscopic nasobiliary drainage; BMI, body mass index; ALT, alanine aminotransferase; AST, aspartate aminotransferase; TBIL, total bilirubin; DBIL, direct bilirubin; CRP, C-reactive protein. Values are expressed as mean ± standard deviation.

## Discussion

AOC is a common and severe infectious disease that occurs in an obstructed biliary system. Patients with AOC benefit from effective antibiotics and technological advances related to the BD, including percutaneous transhepatic-cholangial drainage (PTCD), ENBD, and biliary surgery, and mortality associated with AOC has declined significantly. However, for some patients with severe disease, AOC-induced organ failure is still lethal^29, 30^. The liver is the organ that is initially involved, and functional restoration of the liver will determine the prognosis of patients with AOC. In clinical practice, estimating the prognosis of patients with AOC using liver function tests requires 48–72 h after BD^29, 31^. Determining the probable trend of liver restoration as early as possible may be important for timely adjustment of the treatment plan.

Essentially, AOC is a specific inflammation that occurs in the setting of biliary obstruction. Predictably, regulatory factors of inflammatory signaling may play a crucial role in this disease. The SOCS family is known to be regulators of cytokine receptor signaling and was studied by us. In this study, we showed that there were changes in SOCS1 and SOCS3 expression in AOC animal models. SOCS3 has been reported to be involved in both cholestasis and inflammation^14, 32^. SOCS1 expression increases after LPS stimulation in hepatocytes^33^, while its function remains obscure during cholestasis. Here, we showed their expression changes during this special pathological condition.

Effective BD is more important than antibiotic therapy and it is always regarded as the preferred therapeutic strategy for patients with AOC^29^. We also simulated the BD process in rat models. The results showed that SOCS1, but not SOCS3, regulated the regenerative response and was associated with the AOC prognosis. Both SOCS1 and SOCS3 were reported to effectively regulate liver regeneration after hepatectomy^28, 34^. BD can significantly reduce endotoxin uptake, but the status of cholestasis cannot recover within a short time. Our results showed that SOCS3 expression increased significantly because of biliary obstruction, while that of SOCS1 did not show a similar change (Fig. 1B and D). Although it cannot be ruled out that SOCS3 had a similar function as SOCS1, the unrecovered status of cholestasis provides a possible explanation. Our results indicate that SOCS1 expression was correlated with AOC prognosis, while the correlation between SOCS3 expression and AOC prognosis was less clear.

HGF plays an important role in hepatocyte injury and repair^35, 36^. Our results showed that hepatic HGF levels were not significantly different between rats that survived and those that died. Whether the rats survived or died, the expression of HGF displayed the repair mechanism of liver was activated. Further studies found that a significantly strengthening downstream signaling of HGF arose in rats that survived after BD. To clear the underlying mechanism, we focused on that SOCS1 has a function to control hepatocyte regeneration by regulating HGF signaling^28^. We found that SOCS1 expression decreased in rats that survived. Because SOCS1 expression can be influenced by stress state, such as excessive inflammation stress and oxidative stress^37, 38^, rats that died had a higher SOCS1 expression and they did not show enough activated HGF signaling. We also studied whether aberrant SOCS1 expression regulated hepatocytes proliferation *in vitro*. Both cell proliferation assays and PCNA expression examination showed that silencing of SOCS1 significantly promotes proliferation of hepatocytes (Fig. 3D and I). Our results showed that SOCS1 influenced the results of the contest between liver injury and liver repair. It suggests that SOCS1 may be a prognostic maker for AOC.

However, examining SOCS1 expression requires liver samples. For clinical practice, a liver biopsy is too invasive to perform. Given that peripheral blood can be collected more easily and circulating miRNAs are regarding as important biomarkers for many diseases^16, 39^, we hypothesized the miRNAs can regulate SOCS1 expression or can be regulated by changes in SOCS1 expression. miR-221/222, which can be regulated by Met^17^, were suggested as possible candidates. Our study showed that miR-221/222 expression was modulated by the SOCS1/Met axis *in vitro*, and confirmed the inverse correlation between SOCS1 and miR-221/222 *in vivo*. miR-221/222 were reported to promote proliferation and migration by targeting SOCS1 in basal-like breast cancer^18^. We also investigated whether miR-221/222 reduced LPS-induced SOCS1 upregulation in hepatocytes. However, SOCS1 expression in BRL cells did not show the expected significant decrease after transfection with miR-221 or miR-222 (Fig. 4E), which may be because of LPS stimulation or the difference between human SOCS1 and rat SOCS1. Hence, miR-221/222 were more likely negatively regulated by SOCS1 during the process of AOC. Furthermore, Pearson’s correlation analysis also showed that miR-221 and miR-222 in serum had an inverse correlation with SOCS1 expression, which is similar to the results in liver tissue. Given that both miR-221 and miR-222 have the same sequences in humans and rats, we also explored the potential correlation between circulating miR-221/222 and prognosis of AOC patients. The results suggested that lower expression of circulating miR-221 and miR-222 after ENBD was associated with delayed restoration of liver function. It’s worth noting that the interesting difference arose at 6 hours after ENBD. C-reactive protein (CRP) is a frequently used biomarker for clinical severe sepsis. We also examined CRP level at 6 hours after ENBD. However, the result showed that there was no significant difference between good recovery group and bad recovery group (Table. 2). A recent study verified that patients with spontaneous recovery from acute liver failure had significantly higher serum miR-221 levels than patients who did not recover^40^. More interestingly, miR-221 overexpression has been reported to accelerate hepatocyte proliferation during liver regeneration^41^. Consistent with previous studies, our results suggest that circulating miR-221/222 could reflect SOCS1 expression levels in the liver and indirectly predict the prognosis of patients with AOC.

In summary, our results showed that SOCS1 regulates hepatic regenerative response and that it is a prognostic maker during acute obstructive cholangitis. Circulating miR-221 and miR-222, which are indirectly regulated by SOCS1, may be potential serological biomarkers for predicting the outcome for patients with acute obstructive cholangitis.

## Materials and Methods

### Animal model

Male Wistar rats weighing 300–350 g were purchased from Shanghai SLAC Laboratory Animal Co., Ltd. (Shanghai, China). All procedures were approved by the Ethics Committee of Zhejiang University and conformed to the Care and Use of Laboratory Animals Guide published by the US National Institutes of Health (NIH Publication No. 85–23, revised 1996). The animal study was divided into two parts.

#### Experiment 1

Rats were randomly assigned to the following four groups (n = 6 each): sham, lipopolysaccharide (LPS), bile duct ligation (BDL), and acute obstructive cholangitis (AOC) groups. In the BDL and AOC groups, the distal common bile ducts were dissociated and ligated with 6-0 silk. Epidural catheters (diameter, 0.9 mm) were inserted into the proximal bile ducts and the catheters long enough to reach the skin, as previously described^25^. For intra-bile duct infusion, 0.2 ml of saline or LPS (2 mg/ml, purified from *Escherichia coli* O111:B4, Sigma, St Louis, MO, USA) was injected to proximal bile ducts through the catheter. After 0.1 ml air was injected, the catheter was sealed with a sealing cap and the abdominal cavity was closed using silk sutures. Rats in the sham group underwent a sham operation with intraperitoneal saline infusion and rats in the LPS group underwent a sham operation with intraperitoneal LPS infusion. These rats were euthanized 12 h after the surgery, and blood and tissue samples were harvested from the euthanized animals. Blood samples was centrifuged and serum was stored at −80 °C before the analysis. Liver fractions were snap-frozen in liquid nitrogen and then stored at −80 °C as samples.

#### Experiment 2

Forty-five rats that were in the AOC animal model group were divided as follows: biliary drainage group (BD, n = 30) and no biliary drainage group (NBD, n = 15). On the basis of the AOC model in Experiment 1, right jugular vein catheterization was performed after BDL surgery, according to previously described protocols^25, 42^. In the BD group, the catheter sealing caps were removed and bile was allowed to flow out from catheters at 12 h after LPS infusion. However, in the NBD group, the catheter sealing caps remained closed. All surviving rats underwent a secondary laparotomy 18 h after the first laparotomy and the caudate lobes of the liver were excised and analyzed. Blood samples were also taken through the jugular vein catheter. All surviving rats remained under strict observation and their time of death was recorded during a 72-h observation period after LPS infusion. The rats in the BD group were divided based on their survival during the observation period, as follows: BD-survival and BD-death group. Serum samples and tissues samples were prepared in accordance with the aforementioned criteria.

### Evaluation of liver function

Serum levels of aspartate aminotransferase (AST), alanine aminotransferase (ALT), and total bilirubin (TBIL) were quantitated by an Automated Chemical Analyzer (Dimension RxLMax HM, Siemens, Germany) to evaluate the degree of liver injury.

### RNA extraction and polymerase chain reaction

Total RNA was isolated from samples using TRIzol (Invitrogen) and reverse transcribed into cDNA using the High-Capacity cDNA Reverse Transcription Kit, according to the manufacturer’s instructions. Quantitative real-time PCR was performed using SYBR Green PCR Master Mix and the ABI 7500 Real-time PCR system (Applied Biosystems). Primers sequences used are provided in Table 3. β-actin was used as an endogenous control. The expression levels of miR-221/222 were normalized to U6 RNA (tissue samples) and spiked C. *elegans* miR-39 mimic (serum samples)^43^. Specific primers for miR-221/222, U6 and C. *elegans* miR-39 RNA were obtained from RiboBio (Guangzhou, China). PCR was performed according to the manufacturer’s instructions. All assays were performed three times. Relative expression levels were then determined using the 2^−ΔΔCt^ Method.

**Table 3:** Primer sequences for PCR assay.

Name	Symbol	Forward (5′-3′)	Reverse (5′-3′)
β-actin	ACTB	ACACCCGCCACCAGTTCG	CCCACGATGGAGGGGAAGA
SOCS1	SOCS1	CCTTCGACTGCCTCTTCGAG	AGTCACGGAGTACCGGGTTA
SOCS2	SOCS2	CCGACTAACCTGCGGATTGA	CTGTCCGTTTGTCCTTGCAC
SOCS3	SOCS3	CCCCGCTTTGACTGTGTACT	GTACCAGCGGGATCTTCTCG
SOCS4	SOCS4	GTGTCCAGCCGTGTGTCATA	TTCCCATCTGGGCTTGTTCC
SOCS5	SOCS5	TGGACATGAACGCCAACAGA	GGCACAGTTTTGGTTCCGTC
SOCS6	SOCS6	TTTAGACGACGGGATGTGCC	GGCTCTGCAACATGACTCCT
SOCS7	SOCS7	CTTGGAATCGGAGGCCGAAA	CCAAGTCCTGCAGCGGAAAG
Cis	Cish	CAACACCTGTGTCGGCTAGT	AACGGGTACTGTCGGAGGTA
PCNA	PCNA	CAACTTGGAATCCCAGAACAGGAG	TAAGGTCCCGGCATATACGTGC
cyclin D1	CCND1	GCCATCCATGCGGAAAATC	AGAGACAAGAAACGGTCCAGGT
Bax	Bax	GGGATGGCCTCCTTTCCTAC	CTTTCCCCGTTCCCCATTCA
Bcl2	Bcl2	AGCATGCGACCTCTGTTTGA	TCACTTGTGGCCCAGGTATG

### Western blot analysis

On a 10% SDS–PAGE gel, 20 μg total protein was electrophoresed, transferred onto to a polyvinylidene fluoride membranes, blocked, incubated with primary antibody (anti-SOCS1, Cell Signaling Technology, Boston, MA, USA; anti-SOCS3, Abcam Biotechnological Co., Cambridge, UK; anti-Met, Proteintech, Rosemont, IL, USA; anti-phospho-Met, Cell Signaling Technology; anti-AKT, Proteintech; anti-phospho-AKT, Cell Signaling Technology; anti-β-actin, Abcam Biotechnological Co; anti-PCNA, Santa Cruz.) and then with horseradish peroxidase-conjugated secondary antibody. Immunoreactive bands were visualized using a chemiluminescence solution. β-actin was employed as an endogenous control.

### Immunohistochemistry staining

PCNA expression was detected immunohistochemically using paraffin-embedded specimens from different groups. After deparaffinization and rehydration of the sections, endogenous peroxidase activity was blocked using 0.3% hydrogenperoxide. The sections were incubated with primary anti-PCNA antibody (Santa Cruz) overnight at 4 °C, followed by incubation with appropriate horseradish peroxidase-conjugated secondary antibody for 1.5 h. After a thorough washing, the sections were developed in 3,3′-diaminobenzidine and counterstained with hematoxylin. Each stained sample was observed under high power magnification (×200).

### Caspase 3 activity detection

Caspase 3 activity in cells proteins was detected by the Caspase 3 activity assay kit (Beyotime, Nanjing, China), according to the manufacturer’s protocol. The absorbance was measured at 405 nm, and the relative activity of Caspase 3 was determined.

### Enzyme-linked immunosorbent assay

HGF was measured in liver tissue after cell lysis. A ELISA kit (eBioscience&Bender, San Diego, CA, USA) was used according to the manufacturer’s instructions.

### Cell culture

The rat hepatic cell line BRL was obtained from the Chinese Academy of Sciences Shanghai Branch Cell Bank (Shanghai, China). The cells were cultured in Dulbecco’s modified Eagle’s medium supplemented with 10% fetal bovine serum (FBS), 100 IU/ml penicillin, and 100 ug/ml streptomycin at 37 °C, 5% CO_2_.

### Plasmids, RNA oligonucleotides and transfection

To increase the expression of SOCS1 in BRL cells, rat SOCS1 coding sequences was cloned into pcDNA3.1 vector (Invitrogen Life Technologies). Total RNA was isolated from rat liver tissue and cDNA was synthesized using the PrimeScript reagent kit (Applied Biosystems, Foster City, CA, USA) according to the manufacturer’s instructions. The primers for PCR amplification for sequences of SOCS1-ORF were as follows: 5′-CCAAGCTTATGGTAGCACGTAACCAGG-3′ and reverse: 5′-CCCTCGAGTCAGATCTGGAAGGGGAAGG-3′. The PCR products were purified and cloned into the HindIII/XhoI sites of vector. All the constructs were confirmed by DNA sequencing. At 80–90% confluence, cells were transfected with pcDNA3.1-SOCS1 or empty vectors using lipofectamine 2000 (Invitrogen, Carlsbad, CA, USA).

siRNAs targeting SOCS1 were obtained from RiboBio (Guangzhou, China) and sequences were 5′-GCAUUCGUGUGCACUUCCA-3′. Other RNA oligonucleotides (including miR-221, anti-miR-221, miR-222, anti-miR-222, miR control and siRNA control) was also obtained from RiboBio. Twenty-four hours prior to transfection, cells were plated onto a 35-mm dish at 50–60% confluence. Transfection was performed with lipofectamine 2000 according to the manufacturer’s protocol. The transfected cells were resuspended and cultured in regular culture medium for 48–72 h before analysis.

### Cell proliferation assays

BRL cells were transfected with RNA oligonucleotide or plasmid DNA. Fore hours after transfection, equal numbers of viable cells were seeded in 96-well plates for cell proliferation assay. Cell growth was determined using CCK8 kit. In brief, one-tenth volume of CCK-8 was added to each well, and the cells were cultured for another 2 h. Cell density was determined by measuring the absorbance at 450 nm using an enzyme-labeling instrument (BioTek, Winooski, VT, USA).

### Clinical serum samples

Blood samples from 28 patients who had Charcot triad with high TBIL (100–200 umol/L) and underwent ENBD were obtained at the Shaoxing People’s Hospital from January 2013 to December 2015. Informed consent was obtained from patients and the tissue acquisition protocol was approved by the Zhejiang University Institutional Review Board. All methods with relation to human samples were performed in accordance with the relevant guidelines and regulations set by the ethics committee. Liver function was routinely tested before ENBD and at 3 days after ENBD. At 6 hours after ENBD, the blood samples were collected for our study. After centrifugation, serum was collected into RNase/DNase-free tubes and stored at −80 °C. C-reactive protein (CRP) in serum was measured by kit (Jiancheng Bioengineering Institute, Nanjing, China) according to the manufacturer’s instructions. miRNAs in serum was measured in accordance with the aforementioned criteria.

### Statistical analysis

Data were presented as means ± SEM. Statistical significance between two groups was determined using the Student t-test. One-way ANOVA followed by the Tukey–Kramer adjustment were used to examine differences among multiple groups. Clinical characteristics were compared using Kruskal-Wallis test and chi-square test for categorical variables. *P* < 0.05 was considered to be significant. All statistical analyses were conducted by using SPSS 13.0.

## Electronic supplementary material

Supplementary Information
