# Supplementary material for: SOCS1 regulates hepatic regenerative response and provides prognostic makers for acute obstructive cholangitis
Source: Sci Rep. 2017 Aug 25;7:9482. doi: 10.1038/s41598-017-09865-z (PMC5573403; doi:10.1038/s41598-017-09865-z)
Supplement: Supplementary file 1 — Supplementary Information [file 41598_2017_9865_MOESM1_ESM.pdf]

SOCS1 regulates hepatic regenerative response and provides prognostic makers for acute obstructive cholangitis

Jianhua Yu[a], Weiguang Zhang[b], Hongwei Qian[a], Haijun Tang[a], Weiguo Lin[a], Baochun Lu[a#].

[a]Department of Hepatobiliary Surgery, Shaoxing People's Hospital, Shaoxing Hospital of Zhejiang University, Shaoxing, China

[b]Department of Molecular Medicine and Clinical Laboratory, Shaoxing Second Hospital, Shaoxing, China

[#]Correspondence to Baochun Lu [sygd_lbc@126.com].

Supplementary information

Figure 1

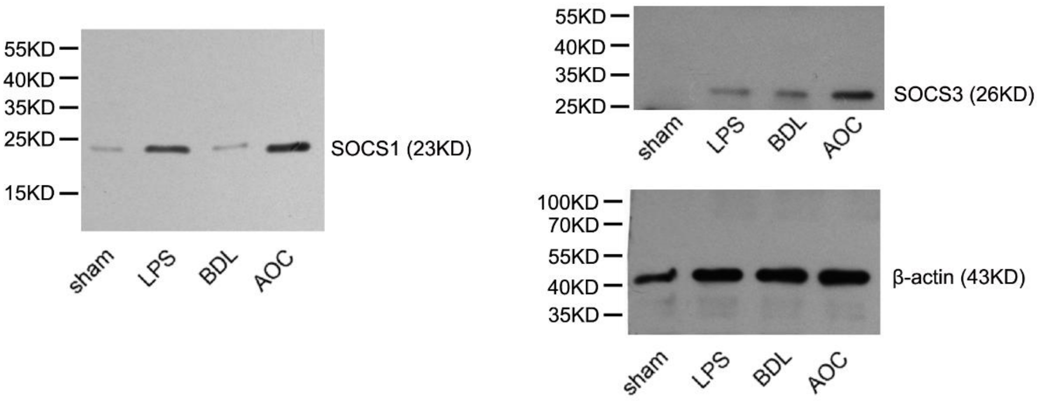

Figure 2

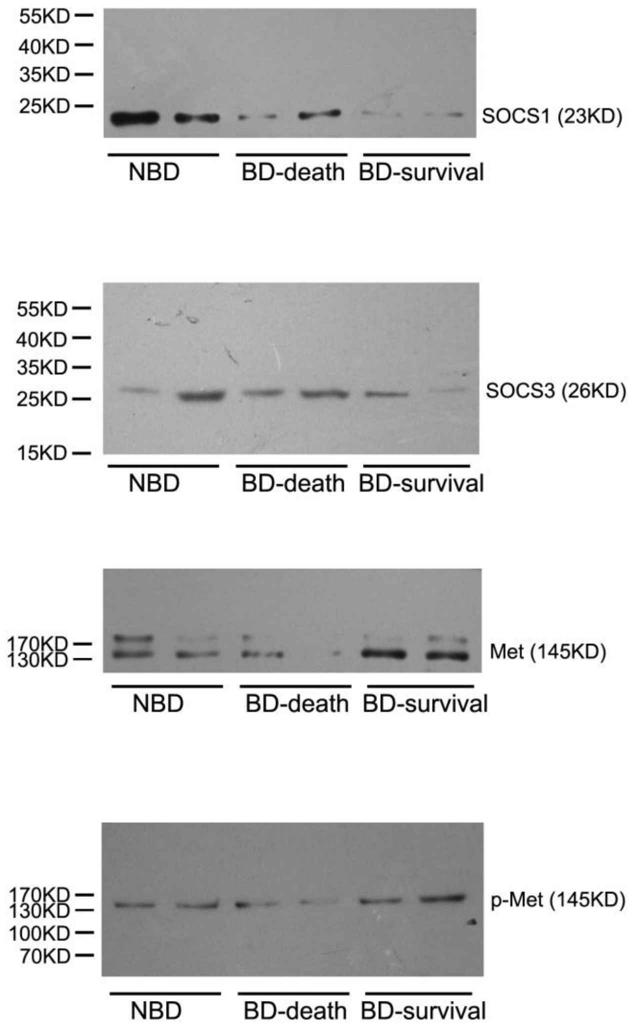
55KD
40KD
35KD
25KD
SOCS1 (23KD)
NBD
BD-death
BD-survival
55KD
40KD
35KD
25KD
15KD
SOCS3 (26KD)
NBD
BD-death
BD-survival
170KD
130KD
Met (145KD)
NBD
BD-death
BD-survival
170KD
130KD
100KD
70KD
p-Met (145KD)
NBD
BD-death
BD-survival

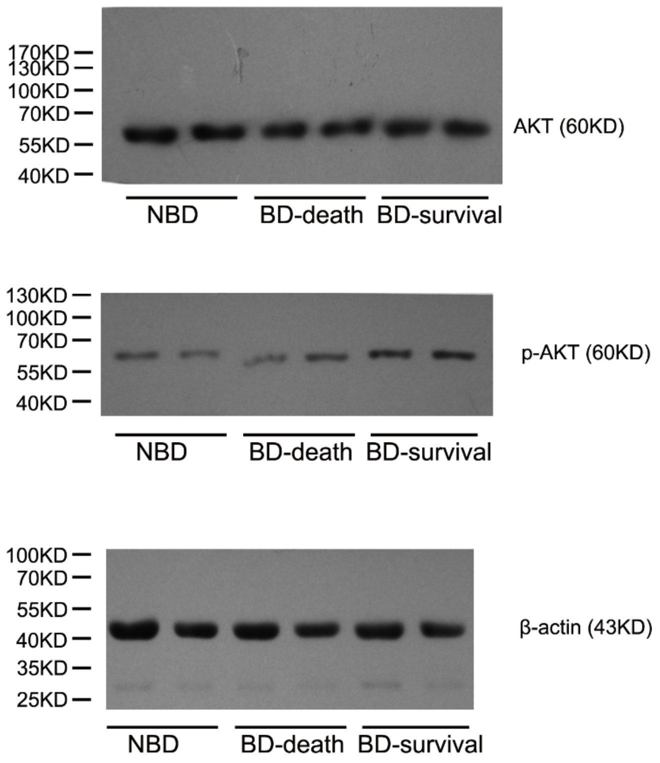

Figure 3

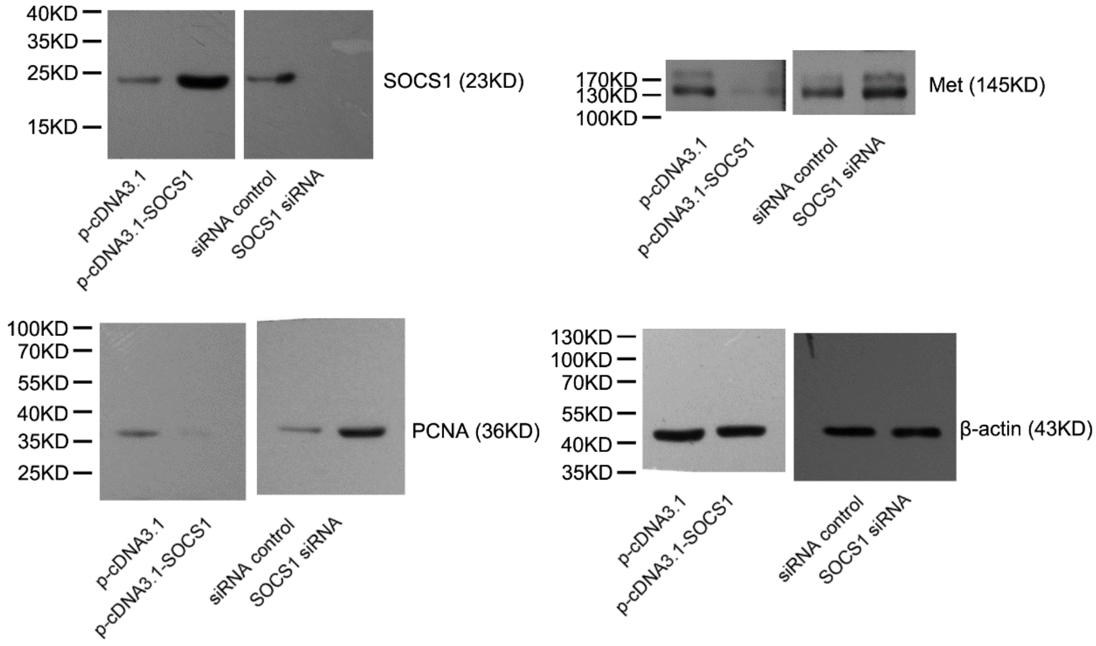

Figure 4

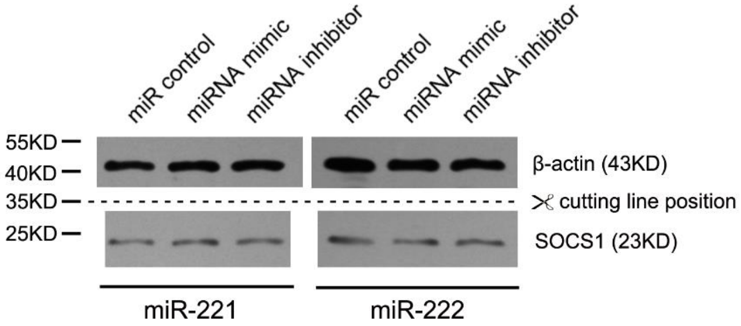
miR control
miRNA mimic
miRNA inhibitor
miR control
miRNA mimic
miRNA inhibitor
55KD
40KD
35KD
25KD
β-actin (43KD)
cutting line position
SOCS1 (23KD)
miR-221
miR-222
